# Nausea and vomiting in early pregnancy: Effects on food intake and diet quality

**DOI:** 10.1111/mcn.12389

**Published:** 2016-11-29

**Authors:** Sarah R. Crozier, Hazel M. Inskip, Keith M. Godfrey, Cyrus Cooper, Sian M. Robinson, SWS Study Group

**Affiliations:** ^1^ MRC Lifecourse Epidemiology Unit, Southampton General Hospital University of Southampton Southampton UK

**Keywords:** cohort study, diet, food frequency questionnaire, nausea, pregnancy, vomiting

## Abstract

Experiences of nausea and/or vomiting in pregnancy (NVP) vary greatly, but the paucity of studies with pre‐pregnancy dietary data mean that little is known about the effects of NVP on diet. Using an administered food frequency questionnaire, diet was assessed before pregnancy and at 11 and 34 weeks' gestation in 2270 participants in a UK birth cohort study (Southampton Women's Survey). Experience of NVP in early pregnancy was graded as none, mild, moderate, or severe. Participants reported their level of food consumption as more, the same, or less than before pregnancy. “Prudent” diet scores (derived using principal component analysis) were used to describe participants' diet quality before, in early and late pregnancy.

In early pregnancy, 89% of women were nauseous, although most commonly, the NVP experienced was mild (48%) or moderate (30%); 11% had severe NVP. A total of 39% of women reported an increase in their level of food intake in early pregnancy; 34% reported a reduction. Increasing severity of nausea was associated with changes in intake of a range of foods, most notably reduced consumption of vegetables, tea/coffee, rice/pasta, breakfast cereals, beans/pulses and citrus fruits/fruit juices and increased consumption of white bread, and soft drinks. Increasing severity of nausea was also associated with decreasing prudent diet score from before to early pregnancy, such that women with severe nausea had prudent diet scores 0.29 SDs lower than those with no nausea (*P* < 0.001). However, this was transient as NVP was not related to change in diet quality from before to late pregnancy.

## 1 INTRODUCTION

Nausea and vomiting in pregnancy (NVP) is common with reported rates ranging from 35% to 91% of pregnancies (Einarson, Piwko, & Koren, 2013). Symptoms range from mild nausea to the serious condition hyperemesis gravidarum (Coad, Al‐Rasasi, & Morgan, 2002; Ebrahimi, Maltepe, & Einarson, 2010). Experiences of NVP vary greatly, but it typically begins around 4–6 weeks gestation, peaks between 8 and 12 weeks, and then diminishes so that by 20 weeks, a markedly reduced number of women suffer from it (Weigel & Weigel, 1989; Flaxman & Sherman, 2000; Patil, Abrams, Steinmetz, & Young, 2012).

Previous analyses in the UK Southampton Women's Survey (SWS) cohort (Crozier, Robinson, Godfrey, Cooper, & Inskip, 2009) demonstrated little overall change in dietary quality from before to early pregnancy but did not evaluate the changes according to NVP or consider changes in energy intake. Relatively little is known about the effects of NVP on diet in pregnancy, although a frequent assumption is that NVP causes a reduction in appetite and reduced food intake. The limited cross‐sectional evidence comparing NVP and reported dietary intakes reveals inconsistent associations. In a recent Norwegian study (Chortatos et al., 2013), women with NVP had slightly higher intakes of fruit and vegetables and more noticeably higher intakes of sugar‐containing soft drinks than other pregnant women. In a smaller Finnish study (Latva‐Pukkila, Isolauri, & Laitinen, 2010) women with NVP ate less meat and somewhat fewer vegetables than other pregnant women, whereas an ecological study across 21 countries (Pepper & Craig Roberts, 2006) suggested that high rates of NVP are associated with high intakes of meat, milk and eggs, and low intakes of cereals and pulses. Some of the inconsistencies in findings may be due to the use of cross‐sectional data, and reporting differences among women whose diets have changed in early pregnancy. However, none of these studies had measures of pre‐pregnancy diet to enable description of changes in diet resulting from NVP.

The present study describes the effects of NVP on change in diet in a cohort of 2270 women whose diets were assessed before and during pregnancy. This provides a valuable opportunity to evaluate change in diet in pregnancy in response to NVP. We consider reported changes in the amount of food and types of foods consumed in early pregnancy, as well as effects on diet quality.

**Key messages**

- Experience of nausea and vomiting in pregnancy is very common; nevertheless women who have mild or moderate nausea in early pregnancy are more likely to report an increase in their level of food intake in early pregnancy than a reduction from pre‐pregnancy levels.
- Women with severe nausea and vomiting in pregnancy much more commonly report a reduction in level of food intake in early pregnancy than an increase.
- Increasing severity of nausea is associated with reduced consumption of vegetables, tea and coffee, rice and pasta, breakfast cereals, beans and pulses, citrus fruit and fruit juices, and increased consumption of white bread, and soft drinks in early pregnancy compared with pre‐pregnancy; these changes overall result in reduced dietary quality.
- This effect is transient as nausea and vomiting in pregnancy is not related to change in diet quality from before to late pregnancy.

## 2 PARTICIPANTS AND METHODS

### 2.1 The Southampton Women's Survey

The SWS is a prospective cohort study in which the diet, body composition, physical activity, and social circumstances of a large group of non‐pregnant women aged 20–34 years living in the city of Southampton, UK, were characterized. Details of the study have been published previously (Inskip et al., 2006). Women were recruited through General Practices across the city between April 1998 and December 2002. Each woman was invited to take part by letter, followed by a telephone call during which an interview date was arranged. A total of 12,583 women agreed, representing 75% of all women contacted. Trained research nurses visited each woman at home and collected information about her health, diet, and lifestyle, as well as taking anthropometric measurements. Women who subsequently became pregnant with singleton fetuses were followed throughout pregnancy; detailed interviews were conducted at 11 and 34 weeks' gestation. The growth and development of the SWS children have been assessed at a number of stages in infancy and childhood, and the children continue to be followed‐up. The SWS was approved by the Southampton and South West Hampshire Local Research Ethics Committee (307/97, 153/99w, 005/03/t, and 06/Q1702/104), and all participants gave written informed consent to be included.

### 2.2 Data collection

Details of mothers' parity and educational attainment (defined in six groups according to highest academic qualification) were obtained at the pre‐pregnancy interview. Height was measured with a portable stadiometer (Harpenden; CMS Weighing Equipment Ltd, London, UK) to the nearest 0.1 cm with the head in the Frankfort plane. Weight was measured using calibrated electronic scales (Seca, Hamburg, Germany) to the nearest 0.1 kg (after removal of shoes and heavy clothing or jewellery). These measurements were used to calculate body mass index (BMI). Before, in early and late pregnancy, food intake over the preceding 3 months was assessed using the same validated interviewer‐administered food frequency questionnaire (FFQ) (Robinson, Godfrey, Osmond, Cox, & Barker, 1996; Crozier, Inskip, Godfrey, & Robinson, 2008); prompt cards were used to ensure standardized responses to the FFQ. Energy intake from the FFQ was computed from portion weights and nutrient content of the foods consumed (Holland, Unwin, & Buss, 1988; Holland, Unwin, & Buss, 1989; Holland, Unwin, & Buss, 1991a; Holland et al., 1991b). Among women who became pregnant, smoking status was ascertained. Experience of NVP was assessed at the 11 week interview and graded as none, mild (nausea only), moderate (sometimes sick), or severe (regularly sick, cannot retain meals); women with hyperemesis were included within the “severe” category. Participants were asked to compare their overall level of food consumption since becoming pregnant as “more than,” “the same,” or “less than” before pregnancy. Women who described their level of food intake as having changed were asked to give the main reason for the change.

### 2.3 Principal component analysis

There were 98 foods and non‐alcoholic beverages listed on the FFQ. These were combined into 48 food groups on the basis of similarity of nutrient composition and comparable usage. For example, carrots, parsnips, swedes, and turnips were combined to form a root vegetables group; bacon, ham, corned beef, meat pies, and sausages were combined in the processed meats group. Principal component analysis (PCA) is a statistical technique that produces new variables that are uncorrelated linear combinations of the dietary variables with maximum variance (Joliffe & Morgan, 1992). A total of 12,572 women had a dietary assessment at the pre‐pregnancy interview; PCA was performed on reported frequencies of consumption of the 48 foods and food groups, based on the correlation matrix to adjust for unequal variances of the original variables. The first principal component identified a pattern that was consistent with dietary recommendations (Crozier et al., 2009). From this pattern “prudent” diet scores before pregnancy were calculated by multiplying the coefficients from the PCA by each woman's standardized reported frequencies of pre‐pregnancy consumption and were interpreted as a measure of diet “quality”. The prudent pattern was also identified as the first pattern in the PCA of the early and late pregnancy data (Crozier et al., 2009). However, in order to be able to describe change in prudent diet scores, “applied” scores (Crozier et al., 2009) were calculated in pregnancy. These used the coefficients from the pre‐pregnancy PCA multiplied by the early or late pregnancy frequencies of consumption (which were standardized using the means and SDs of the pre‐pregnancy frequencies of consumption). By using an identical scale the prudent diet scores at the two‐time points can be compared directly, and change in score can be assessed. Both the pre‐pregnancy and the applied pregnancy prudent diet scores were divided by the SD of the pre‐pregnancy prudent diet score, so that comparisons could be made in terms of change in SD units. A total of 2270 women completed an FFQ in early pregnancy and these women comprise the analysis sample. Of these, 2057 women completed a late pregnancy FFQ.

### 2.4 Statistical analysis

Summary statistics are presented as mean (SD) or median (IQR) for continuous variables and percentages for categorical variables. *T*‐tests (for normally distributed continuous variables), Mann–Whitney *U*‐Tests (for non‐normally distributed continuous variables), and Chi‐squared tests (for categorical variables) were used to compare the distributions of maternal characteristics between those in the analysis sample and those who notified the SWS of their pregnancy after 11 weeks' gestation; BMI was log‐transformed to normality. Maternal height, pre‐pregnancy BMI, education, parity, age in early pregnancy, smoking in pregnancy, and offspring sex were considered as characteristics associated with NVP; Spearman correlation coefficients were used to test ranks of NVP across continuous variables, and Mann–Whitney *U*‐Tests to compare ranks of NVP across binary variables.

Based on a review of the literature on NVP, we used the directed acyclic graph approach (Greenland, Pearl, & Robins, 1999) to select suitable confounders for linear regression models to assess the effect of nausea on change in diet (Supplementary Figure 1); the confounders identified by the direct acyclic graph were educational attainment, BMI, age, parity, and smoking in early pregnancy.

We considered NVP as a predictor of change in intakes of foods/food groups, energy and the prudent diet score from before to early pregnancy, using linear regression models. NVP was included as a categorical variable with “no nausea” as the reference category. Changes in the prudent diet score from before to late pregnancy were also considered to enable us to examine the consistency of the effects of NVP on changes in diet quality later in pregnancy. Analyses were performed using Stata 14.0 (StataCorp, 2015).

## 3 RESULTS

The characteristics of the 2270 women studied and the 597 who did not have early pregnancy interviews are summarized in Table 1. In early pregnancy, the majority of women were nauseous (89%), although most commonly, the NVP experienced was mild (48%) or moderate (30%); 11% had severe NVP symptoms. Despite the high prevalence of NVP, women were marginally more likely to report an increase in their level of food intake (39%) than a reduction (34%) in early pregnancy, compared with the pre‐pregnant period. Compared with the 597 participants who did not have early pregnancy interviews, the 2270 women in the current study were taller (*P* = 0.003), had a higher level of education (*P* < 0.001), were more likely to be nulliparous (*P* < 0.001), likely to have taken less time to conceive (*P* = 0.002), had higher prudent diet scores before pregnancy (*P* = 0.01), and had lower energy intakes (*P* = 0.002). However, there were no differences in pre‐pregnancy BMI (*P* = 0.87), age in early pregnancy (*P* = 0.85) or infant sex (*P* = 0.85) between the two groups.

**Table 1:** Descriptive statistics for pregnant women in the Southampton Women's Survey

Characteristic	2270 women with early pregnancy interview	597 women with no early pregnancy interview	*P*‐value	*n*
Height (m) a	163.4 (6.4)	162.5 (6.7)	0.003	2854
Pre‐pregnancy BMI (kg/m^2^) b	24.3 (21.9–27.5)	24.2 (21.8–27.7)	0.87	2845
Pre‐pregnancy ≥ A‐level (%)	60.0	51.5	<0.001	2860
Nulliparous (%)	51.2	41.7	<0.001	2865
Time to conception (years)^b^	1.5 (0.7–2.8)	1.8 (0.8–2.9)	0.002	2817
Age in early pregnancy (years) a	30.0 (3.7)	30.0 (4.1)	0.85	2867
Gestation in early pregnancy (weeks) a	11.9 (0.8)	–	–	2220
Smoking in early pregnancy (%)	14.3	–	–	2259
Early pregnancy NVP c (%)	–	–	–	2269
None	10.9	–	–	–
Mild	47.8	–	–	–
Moderate	30.4	–	–	–
Severe	10.9	–	–	–
Early pregnancy reported change in level of food intake (%)	–	–	–	2266
Less	33.7	–	–	–
The same	27.7	–	–	–
More	38.6	–	–	–
Pre‐pregnancy prudent diet score (SDs) a	0.06 (0.96)	−0.05 (1.01)	0.01	2866
Early pregnancy prudent diet score (SDs) a	0.06 (0.93)	–	–	2270
Early – pre‐pregnancy prudent diet score (SDs) a	0.00 (0.72)	–	–	2270
Pre‐pregnancy energy intake (kcal) b	2015 (1696–2422)	2085 (1741–2557)	0.002	2866
Early pregnancy energy intake (kcal) b	2089 (1732–2481)	–	–	2270
Early – pre‐pregnancy energy intake (kcal) b	52 (−292–416)	–	–	2270
Female offspring (%)	47.9	52.1	0.85	2816

a Mean (Standard deviation)

b Median (Interquartile range)

c Nausea and vomiting in pregnancy

Characteristics of women in the study were compared across the four NVP groups (Table 2). There was a difference in nausea severity between the two parity groups; among women in their first pregnancy 13% had no NVP and 8% had severe NVP, whereas among women in their second or subsequent pregnancy 9% had no NVP and 13% had severe NVP. Women with more nausea tended to be slightly younger; the average age of women with no NVP was 30.4 years, whereas the average age of women with severe NVP was 29.3 years. Women with more severe nausea had lower levels of education and tended to have slightly higher BMI. There was no association between level of nausea and maternal height, smoking in early pregnancy, or offspring sex.

**Table 2:** Maternal descriptive statistics by NVP a group

Characteristic	None	Mild	Moderate	Severe	*P*‐value d	n
Height (m) b	163.3 (6.0)	163.7 (6.3)	163.3 (6.7)	162.9 (6.5)	0.17	2261
Pre‐pregnancy BMI (kg/m^2^) c	24.3 (21.8–26.8)	24.1 (21.8–27.2)	24.5 (22.0–27.7)	24.5 (22.2–29.2)	0.01	2253
Educational attainment (%)	–	–	–	–	0.005	2263
<A‐levels	11%	45%	32%	12%	–	–
≥A‐levels	11%	50%	30%	10%	–	–
Parity (%)	–	–	–	–	<0.001	2267
0	13%	48%	30%	8%	–	–
1+	9%	47%	30%	13%	–	–
Age in early pregnancy (years) b	30.4 (3.9)	30.3 (3.6)	29.7 (3.8)	29.3 (3.8)	<0.001	2269
Smoking in early pregnancy (%)	–	–	–	–	0.14	2259
No	11%	49%	29%	11%	–	–
Yes	13%	40%	36%	11%	–	–
Offspring sex (%)	–	–	–	–	0.69	2219
Males	12%	45%	31%	11%	–	–
Females	10%	50%	30%	11%	–	–

a Nausea and vomiting in pregnancy

b Mean (Standard deviation)

c Median (Interquartile range)

d From Spearman correlation or Mann–Whitney *U‐*Test

**Figure 1:**
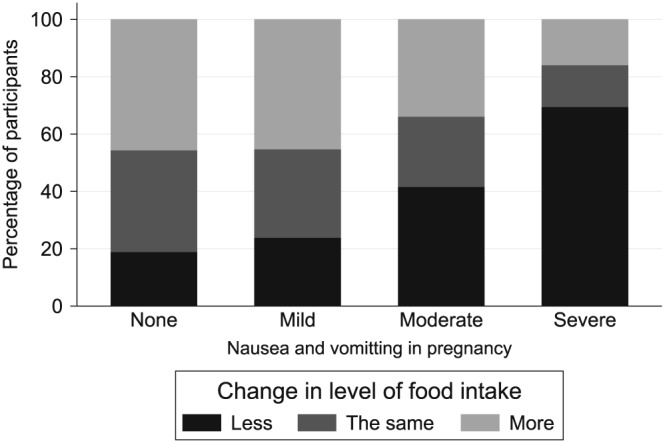
Reported change in levels of food intake in early pregnancy compared with before pregnancy, according to experience of nausea and vomiting in pregnancy (*n* = 2266)

The percentages of women reporting changes in their level of food intake are shown according to severity of nausea experienced in Figure 1. Among women with mild or moderate NVP, 24% and 42% of women reported a reduced level of intake in early pregnancy respectively; among women with mild or moderate NVP, 45% and 34% of women reported an increased level of food intake in early pregnancy, respectively. Thus for the majority of women who had none, mild, or moderate nausea in early pregnancy, increases as well as decreases in the level of food consumption were similarly common (41% of these women increased, compared with 29% decreased). However, these proportions were markedly different among the women whose NVP was severe, for whom a reduction in level of intake was much more common (70%) than an increase (16%) in early pregnancy.

The main reason given for change in level of intake from before pregnancy was examined in relation to the severity of NVP experienced in early pregnancy (Table 3). A total of 764 women reported that their overall level of food intake was reduced in early pregnancy. The 47 women with no NVP mainly ate less because they felt less hungry (72%), whereas the 717 women with mild, moderate or severe NVP mainly ate less because of nausea/sickness (66%). A total of 875 women reported that their overall level of food intake was increased in early pregnancy. The 113 women with no NVP mainly ate more because they felt hungrier (76%). However, among the 762 women with mild, moderate, or severe NVP, roughly equal proportions of women ate more because they felt hungrier (48%) and because of the nausea/sickness experienced (42%).

**Table 3:** Principal reason given for eating more or less in early pregnancy according to experience of NVP a

	Reasons for eating less in pregnancy (*n* = 764)			Reasons for eating more in pregnancy (*n* = 875)		
NVP	Because feel less hungry	Because of nausea/sickness	Other reason	Because feel more hungry	To prevent feeling sick	Other reason
None	34 (72%)	0 (0%)	13 (28%)	86 (76%)	7 (6%)	20 (18%)
Mild	98 (38%)	143 (55%)	18 (7%)	256 (52%)	183 (37%)	51 (10%)
Moderate	90 (31%)	181 (63%)	16 (6%)	93 (40%)	120 (52%)	20 (9%)
Severe	20 (12%)	148 (87%)	3 (2%)	18 (46%)	17 (44%)	4 (10%)
Total	242 (32%)	472 (62%)	50 (7%)	453 (52%)	327 (37%)	95 (11%)

a Nausea and vomiting in pregnancy

Table 4 shows associations between change in food/food groups from before to early pregnancy and nausea as a 4‐level variable (as the exposure), adjusted for confounders. There were changes in reported consumption of a range of foods in early pregnancy. The associations between nausea and vegetable intake are particularly notable; women with no NVP decreased their total vegetable consumption (calculated by summing consumption of salad vegetables, green vegetables, root vegetables, other vegetables, tinned vegetables, and vegetable dishes) by 0.9 portions per week, whereas women with severe NVP decreased their total vegetable consumption by 5.6 portions per week. The association between nausea and tea and coffee intake is also strong; women with no NVP drank tea and coffee on six fewer occasions per week in early pregnancy compared with before pregnancy, whereas women with severe NVP drank tea and coffee on 11.7 fewer occasions per week in early pregnancy compared with before pregnancy. Participants with greater levels of nausea also tended to eat rice and pasta, breakfast cereals, beans and pulses, citrus fruit and fruit juices less frequently, and white bread and soft drinks more frequently.

**Table 4:** Mean change in frequency of food consumption from before to early pregnancy according to experience of NVP a ^,^ b (*n* = 2269)

Food or food group	None	Mild	Moderate	Severe
**Rice and pasta (freq/week)**	−0.20	−0.32	−0.67*	−0.64
**White bread (slices/week)**	−0.44	0.41	1.90*	1.16
Wholemeal bread (slices/week)	0.65	0.41	−0.03	−0.53
Quiche and pizza (freq/week)	−0.06	0.03	0.03	−0.08
Yorkshire pudding and savoury pancakes (freq/week)	0.04	0.00	0.05	−0.03
**Breakfast cereals (freq/week)**	1.03	0.74	0.38	0.10*
Cakes and biscuits (freq/week)	0.30	0.64	1.37	1.74
Puddings (freq/week)	0.06	−0.08	−0.04	0.14
Cream (freq/week)	0.02	0.02	0.02	−0.02
Full‐fat milk (pints/day)	0.00	0.01	0.00	0.01
Reduced‐fat milk (pints/day)	0.03	0.03	−0.00	−0.03
Yoghurt (freq/week)	0.07	0.03	−0.29	−0.66
Cheese and cottage cheese (freq/week)	0.14	0.34	0.26	0.04
Eggs and egg dishes (freq/week)	−0.12	−0.13	0.03	−0.06
Full‐fat spread (freq/week)	0.81	0.46	0.52	0.39
Reduced‐fat spread (freq/week)	−0.38	0.10	0.58	0.19
Cooking fats and salad oils (freq/week)	−0.04	0.12	−0.17	−0.51
Red meat (freq/week)	0.12	−0.01	0.03	−0.11
Chicken and turkey (freq/week)	−0.02	−0.09	−0.07	−0.13
Offal (freq/week)	−0.16	−0.15	−0.19	−0.15
Processed meat (freq/week)	0.46	0.29	0.13	−0.00
Fish and shellfish (freq/week)	0.08	−0.10	−0.20	−0.25
**Salad vegetables (freq/week)**	−0.09	−0.41	−1.05*	−1.79*
Green vegetables (freq/week)	−0.04	−0.19	−0.30	−0.98
Root vegetables (freq/week)	−0.10	−0.11	−0.07	−0.19
**Other vegetables (freq/week)**	−0.35	−0.70	−1.05*	−1.75*
Tinned vegetables (freq/week)	−0.09	−0.05	−0.10	−0.13
**Vegetable dishes (freq/week)**	−0.26	−0.46	−0.40	−0.72*
**Beans and pulses (freq/week)**	0.17	−0.00	−0.15*	−0.24*
Chips and roast potatoes (large potato/week)	−0.12	−0.03	−0.07	−0.07
Boiled potatoes (large potato/week)	0.04	−0.16	−0.32	−0.20
Crisps (freq/week)	−0.03	0.45	0.59	0.63
Crackers (freq/week)	0.04	0.24	0.67	0.71
**Citrus fruit and fruit juices (freq/week)**	2.81	1.66	1.65	1.01*
Other fruit (freq/week)	1.88	0.93	0.93	0.30
Other fruit juices (freq/week)	0.77	0.68	0.63	0.09
Dried fruit (freq/week)	0.29	0.29	0.08	−0.09
Cooked and tinned fruit (freq/week)	0.07	0.05	−0.01	0.06
Nuts (freq/week)	−0.05	−0.04	−0.03	−0.01
Sugar (tsp/day)	−0.14	−0.35	−0.46	−0.53
Sweet spreads and jam (freq/week)	0.24	0.28	0.42	0.55
Sweets and chocolate (freq/week)	0.72	0.05	0.47	0.45
**Soft drinks (freq/week)**	−1.28	0.17	0.68	2.19*
Diet coke (freq/week)	−0.66	−0.88	−0.88	−0.95
**Tea and coffee (freq/week)**	−6.01	−9.89*	−11.85*	−11.74*
Decaffeinated tea and coffee (freq/week)	0.74	0.10	−0.33	−0.45
Hot chocolate drinks (freq/week)	0.71	0.37	0.37	0.50
Miscellaneous (freq/week)	0.66	0.33	0.43	−0.10

* Different from reference category (no nausea) (*P* < 0.01) in a linear regression model, adjusted for educational attainment, BMI, age, parity, and smoking in early pregnancy

a Foods highlighted in bold have at least one comparison with *P* < 0.01

b Nausea and vomiting in pregnancy

We considered whether the reported changes in food consumption led to change in energy intake and diet quality. Overall, there was a small increase in energy intake in early pregnancy (52 kcal, Table 1). In a univariate analysis, increasing severity of NVP was associated with decreases in energy intake from before to early pregnancy; these decreases were small for the mild and moderate nausea categories but statistically significant for the severe group (adjusted difference = −36 kcal, 95% confidence interval − 114 to 43, *P* = 0.38 for the change in energy intake for women with mild nausea compared with the no nausea group, adjusted difference = −33 kcal, −116 to 49, *P* = 0.43 for women with moderate nausea and adjusted difference = −112 kcal, −212 to −12, *P* = 0.03 for women with severe nausea, *n* = 2269). Controlling for confounders slightly reduced the magnitude of the associations (adjusted difference = −31 kcal, −110 to 48, *P* = 0.44 for women with mild nausea, adjusted difference = −31 kcal, −114 to 52, *P* = 0.46 for women with moderate nausea and adjusted difference = −101 kcal, −202 to 0, *P* = 0.05 for women with severe nausea, *n* = 2236). Restricting the analysis to the 789 women who became pregnant within a year revealed a similar but weaker association (adjusted difference = −8 kcal, −136 to 120, *P* = 0.90 for women with mild nausea, adjusted difference = 10 kcal, −128 to 149, *P* = 0.88 for women with moderate nausea and adjusted difference = −75 kcal, −236 to 87, *P* = 0.36 for women with severe nausea, *n* = 789).

**Figure 2:**
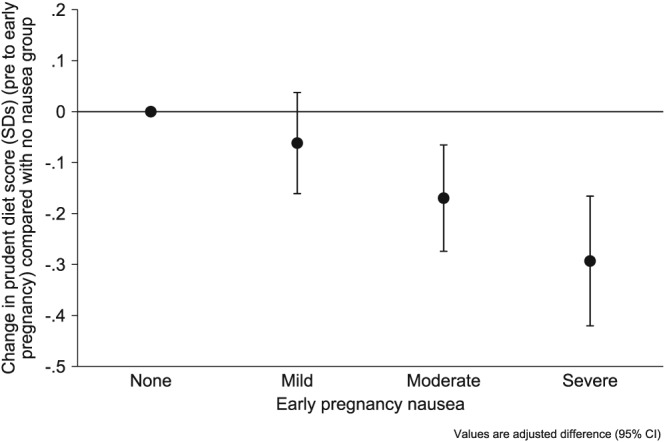
Change in prudent diet in early pregnancy according to experience of nausea and vomiting in pregnancy, adjusted for confounders (*n* = 2236)

As described previously (Crozier et al., 2009), when considering all women, the average prudent diet score did not change from before to early pregnancy (Table 1). There was no graded association between pre‐pregnant prudent diet score and level of nausea experienced, but there was a graded association between NVP and change in prudent diet score in early pregnancy. In a univariate analysis, increased severity of NVP was associated with greater falls in prudent diet score (adjusted difference = −0.08 SD, −0.18 to 0.02, *P* = 0.10 for the change in prudent diet score for women with mild nausea compared with the no nausea group, adjusted difference = −0.19 SD, −0.29 to −0.09, *P* < 0.001 for women with moderate nausea and adjusted difference = −0.33 SD, −0.45 to −0.20*, P* < 0.001 for women with severe nausea, *n* = 2269). Controlling for confounders made little difference to the association (adjusted difference = −0.06 SD, −0.16 to 0.04, *P* = 0.22 for women with mild nausea, adjusted difference = −0.17 SD, −0.27 to −0.07, *P* = 0.001 for women with moderate nausea and adjusted difference = −0.29 SD, −0.42 to −0.17, *P* < 0.001 for women with severe nausea, *n* = 2236). The modelled effect of nausea on change in prudent diet score is illustrated in Figure 2. Restricting the analysis to the 789 women who became pregnant within a year revealed a similar trend across NVP categories (adjusted difference = −0.11 SD, −0.26 to 0.04, *P* = 0.16 for women with mild nausea, adjusted difference = −0.17 SD, −0.34 to −0.01, *P* = 0.04 for women with moderate nausea and adjusted difference = −0.26 SD, −0.45 to −0.07, *P* = 0.009 for women with severe nausea, *n* = 789).

Unlike the changes in diet quality observed in early pregnancy in relation to NVP, there was no association between NVP and change in prudent diet score from before to late pregnancy. In a univariate analysis, increased severity of NVP was not associated with change in prudent diet score from before to late pregnancy (adjusted difference = −0.07 SD, −0.18 to 0.03, *P* = 0.17 for the change in prudent diet score for women with mild nausea compared with the no nausea group, adjusted difference = −0.08 SD, −0.19 to 0.03, *P* = 0.17 for women with moderate nausea and adjusted difference = −0.11 SD, −0.24 to 0.02, *P* = 0.10 for women with severe nausea, *n* = 2056). Controlling for confounders made little difference to the association (adjusted difference = −0.06 SD, −0.16 to 0.05, *P* = 0.29 for women with mild nausea, adjusted difference = −0.04 SD, −0.15 to 0.07, *P* = 0.44 for women with moderate nausea and adjusted difference = −0.05 SD, −0.18 to 0.08, *P* = 0.46 for women with severe nausea, *n* = 2026). The modelled effect of nausea on change in prudent diet score from before to late pregnancy is illustrated in Supplementary Figure 2.

## 4 DISCUSSION

We have used data from a large cohort of women studied before and during pregnancy to examine changes in diet in pregnancy, with a focus on the effects of NVP experienced in early pregnancy. The majority of women experienced some nausea (89%). A total of 11% of women reported severe nausea, defined as “regularly sick, can't retain meals.” Despite the prevalence of NVP, comparable numbers of women described their overall intake in early pregnancy as greater (39%) , compared with less than (34%), the pre‐conception period, and reported increases in food intake in response to NVP were common (48%). Only among women with severe NVP was a reported reduction in level of food intake much more common (70%) than an increase (16%). Average consumption of vegetables and tea and coffee was reduced amongst all women with NVP in early pregnancy, but the reduction was greatest amongst those whose NVP was severe. The changes in foods consumed by women with NVP only resulted in notable changes in energy intake among women with severe nausea, but there were significant graded effects on diet quality such that women with high levels of NVP in early pregnancy were more likely to have a less healthy diet than they had before pregnancy. However, examination of changes in diet quality in late pregnancy showed the changes associated with NVP in early pregnancy were no longer evident, suggesting these changes were transient.

There is wide variability internationally in reported rates of NVP with a systematic review (Einarson et al., 2013) suggesting a median rate of 69% (range 35–91%) and, in six studies where the women with NVP reported the severity (Robertson, 1946; Crystal, Bowen, & Bernstein, 1999; Chou, Avant, Kuo, & Fetzer, 2008; Lacasse, Rey, Ferreira, Morin, & Berard, 2009; Chan et al., 2010; Kramer, Bowen, Stewart, & Muhajarine, 2013), the rates were as follows: mild (40%), moderate (46%), or severe (14%). The prevalence of NVP found in the present study (89%) was at the upper end of the range described in the systematic review, but the distribution of women according to severity of NVP was comparable (53%, 34% and 12%, respectively).

Three previous studies examined the effects of NVP on food intake using cross‐sectional data, two in Scandinavia (Latva‐Pukkila et al., 2010; Chortatos et al., 2013) and one ecological study based on international data (Pepper & Craig Roberts, 2006). There is little consistency in the findings. In the Norwegian study, women with more severe NVP consumed more soft drinks, as in the SWS. The Finnish study found that NVP was associated with lower vegetable consumption (consistent with the SWS), whereas the Norwegian and ecological studies observed a positive association between level of NVP and vegetable intake. The reasons for inconsistency in findings are not clear, although the limitation of the use of cross‐sectional data is likely to be important.

The reported differences in food consumption resulted in differences in diet quality in association with NVP in early pregnancy. Using applied diet pattern scores derived from principal component analysis in this cohort (Crozier et al., 2009), we have previously described little overall change in diet quality from before to early pregnancy. In that study, we did not examine the effects of NVP. In the new analyses reported in the present paper, we have found significant differences in changes in consumption of some foods in response to NVP and, while this only resulted in differences in energy intake in the severe group, there were notable graded differences in the changes in diet quality in early pregnancy among women across all NVP groups.

### 4.1 Strengths and weaknesses

In this study, we interviewed young women both before and twice during pregnancy. A notable strength of the data is that dietary information was collected before pregnancy, thus providing a valuable opportunity to assess dietary change in pregnancy using prospective data. Data were available from a large cohort of women with a good response rate: 75% of the women contacted agreed to take part in the study. The complete cohort of 12,583 non‐pregnant women has been shown to be broadly representative of women of this age group in the UK in terms of smoking and educational profile, although the proportion of white women is higher than the national figure (Inskip et al., 2006). When compared with participants who were also followed through pregnancy but did not have early pregnancy dietary data, the women in the study were taller, better educated, more likely to be nulliparous, likely to have taken less time to conceive and ate a more prudent diet and had a lower energy intake before pregnancy; however, unless the associations between nausea and change in diet in the group studied are different in the remainder of the cohort, it is unlikely that selection bias could explain our findings, but the differences may have implications for their external validity.

A limitation of the SWS data is that there is variability in the time from pre‐pregnancy interview to conception (Table 1), meaning that for some women changes in diet between the period before pregnancy and the early pregnancy interview may include changes that occurred before conception. However, when the analyses were restricted to those who became pregnant within a year of the initial interview, the effect size for the associations between NVP and change in energy intake and prudent diet score were similar to the differences observed for the full sample.

Diet was assessed using an FFQ administered by trained research nurses (Robinson et al., 1996). Although there is a concern that FFQs may be subject to bias (Byers, 2001), they have been shown to reveal similar patterns of diet to those identified using other dietary methods, and with which they are highly correlated (Hu et al., 1999; Khani, Ye, Terry, & Wolk, 2004; Crozier et al., 2008). FFQs have also demonstrated relative stability in the assessment of dietary patterns over time (Hu et al., 1999; Khani et al., 2004; Borland et al., 2008). The estimation of energy intake using self‐reported dietary data is a topic of current concern (Dhurandhar et al., 2015). Although we did not observe large changes in energy intake across the nausea categories in early pregnancy associated with NVP, there may have been small differences in intake that were obscured by measurement error. A further issue is that the food frequency questionnaires we used did not include information about portion size for the majority of foods, and it is possible that changes in portion size from before to early pregnancy could have an impact on our findings. Portion size may be influenced by experience of NVP, or by advice to pregnant women such as that to eat “little and often.” Final considerations are that we relied on women's own assessment of frequency of vomiting, that we did not quantify retention of food consumed (which for some women who were vomiting regularly would have been a key influence on energy absorbed), that the single measure of NVP used does not reflect variation in women's experiences as gestation increases, and that we were unable to explore the effects of hyperemesis specifically as an exposure. However, accepting these limitations, the small differences in energy intake we found, together with the significant variability among the nauseous women in their perceptions of changes in their overall level of food intake in early pregnancy, suggest that the commonly held assumption that NVP causes a reduction in food intake may be most applicable only in women with severe nausea.

In an observational study, it is not possible to determine whether associations are causal. We used a directed acyclic graph approach (Greenland et al., 1999) to identify relevant confounding variables in order to most clearly identify the causal association between NVP and change in diet. However, the possibility of residual confounding cannot be completely eliminated.

### 4.2 Interpretation and implications

Diet in pregnancy is widely recognized as having important consequences for offspring health (Jackson & Robinson, 2001; Englund‐Ogge et al., 2014; Langley‐Evans, 2014). Dietary guidelines in pregnancy highlight the need to consume a varied, balanced diet including plenty of fruit and vegetables, carbohydrates and fibre, as well as protein and dairy foods, and reduce intake of alcohol and caffeinated drinks (Jackson & Robinson, 2001; Eating while you are pregnant, 2002). Women in all NVP groups were successful in reducing their intake of tea and coffee; although, it was those with greater NVP whose intake of tea and coffee decreased the most. On the other hand, the decrease in vegetable consumption across all NVP groups was contrary to dietary guidelines, and the decrease among those with severe NVP was most marked. Other notable changes in diet associated with increased NVP were reduced consumption of rice and pasta, breakfast cereals, beans and pulses, citrus fruit and fruit juices, and increased consumption of white bread and soft drinks.

Pregnancy is often characterized by dietary cravings and aversions. Women with greater NVP tend to experience more aversions and cravings (Coad et al., 2002; Weigel et al., 2011), and it may be that some of the changes in diet we observed in pregnancy were a result of cravings and aversions experienced. The widely reported aversion to tea and coffee (Patil et al., 2012) could be the reason women with greater NVP reported decreased tea and coffee consumption compared with women without NVP. Alternatively, women could be changing their dietary habits to manage their nausea symptoms.

It has been suggested that NVP confers functional advantages by preventing intake of substances that may be harmful to the mother or fetus (Flaxman & Sherman, 2000; Pepper & Craig Roberts, 2006) (the maternal and embryo protection hypothesis), or by decreasing nutrient intake thereby stimulating placental growth (Huxley, 2000) (the placental growth and development hypothesis). This latter hypothesis is based on the assumption of a reduction in appetite and overall energy intake resulting from NVP which may be supported by the current study in which severely nauseous women reduced their energy intake. Also, the change in profile of foods consumed in early pregnancy would have resulted in differences in micronutrient intake in early pregnancy which may be consistent with the placental growth and development hypothesis. An important observation in the present study was that the NVP‐related changes we observed in diet quality did not persist in later pregnancy, at the time of maximal fetal nutrient demand. The transient nature of the changes in diet also underlines the importance of women having an adequate nutritional status before conception and any experience of NVP.

Although most women experience NVP in early pregnancy, there appears to be significant variability among women in their responses to NVP. A more detailed understanding of the reasons for changes in dietary choices may be beneficial for the design of future initiatives to support pregnant women, particularly those whose are more severely affected.

## SOURCE OF FUNDING

Core support for SWS is provided by the UK Medical Research Council and the Dunhill Medical Trust, with adjunctive support from the European Union's Seventh Framework Programme (FP7/2007–2013), project EarlyNutrition and ODIN under grant agreement nos. 289346 and 613977. KMG and CC are supported by the National Institute for Health Research through the NIHR Southampton Biomedical Research Centre.

## CONFLICT OF INTEREST

KMG has received reimbursement for speaking at conferences sponsored by companies selling nutritional products, and is part of an academic consortium that has received research funding from Abbott Nutrition, Nestec and Danone. HMI declares that members of her team have received funding from Danone, Nestec and Abbott Nutrition. CC has received consultancy and honoraria from Alliance for Better Bone Health, Amgen, Eli Lilly, GSK, Medtronic, Merck, Novartis, Pfizer, Roche, Servier, Takeda and UCB. SMR and SRC have no conflicts of interest to declare.

## CONTRIBUTIONS

HMI, KMG, SMR and CC designed the research. SRC performed the statistical analyses. SRC, SMR and HMI drafted the paper. All authors read and approved the final manuscript.

## Supporting information

Supplementary Figure 1 Directed Acyclic Graph for early pregnancy nausea and change in diet (from before to early pregnancy)
Supplementary Figure 2 Change in prudent diet in late pregnancy according to experience of NVP, adjusted for confounders (*n* = 2026)

Supplementary Figure 1 Directed Acyclic Graph for early pregnancy nausea and change in diet (from before to early pregnancy)

Supplementary Figure 2 Change in prudent diet in late pregnancy according to experience of NVP, adjusted for confounders (*n* = 2026)
