# Supplementary figures and images for: Nausea and vomiting in early pregnancy: Effects on food intake and diet quality
Source: Matern Child Nutr. 2016 Nov 29;13(4):e12389. doi: 10.1111/mcn.12389 (PMC5400073; doi:10.1111/mcn.12389)

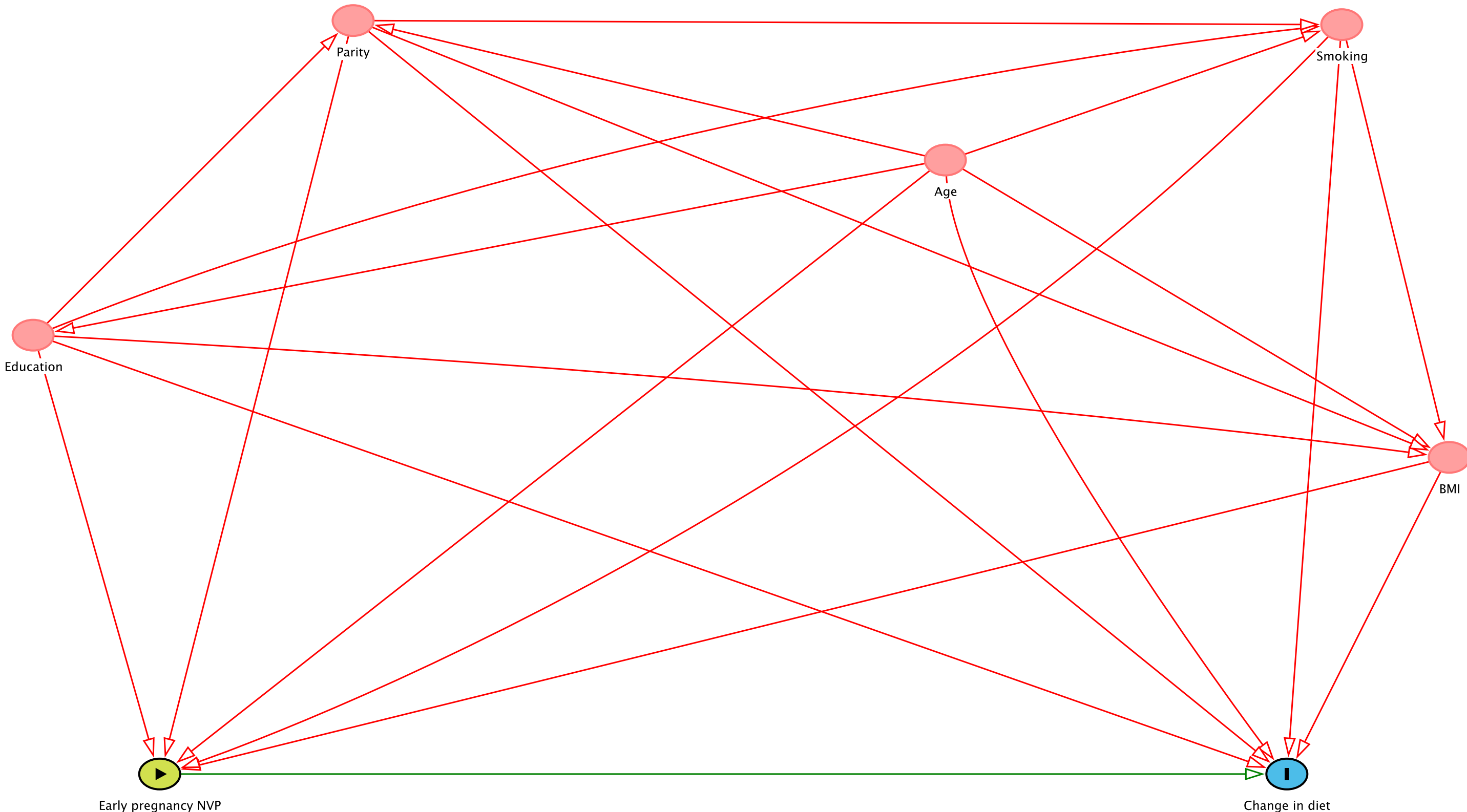

Supplement: Supplementary file 1 — Supplementary Figure 1 Directed Acyclic Graph for early pregnancy nausea and change in diet (from before to early pregnancy) [file MCN-13-e12389-s001.pdf]

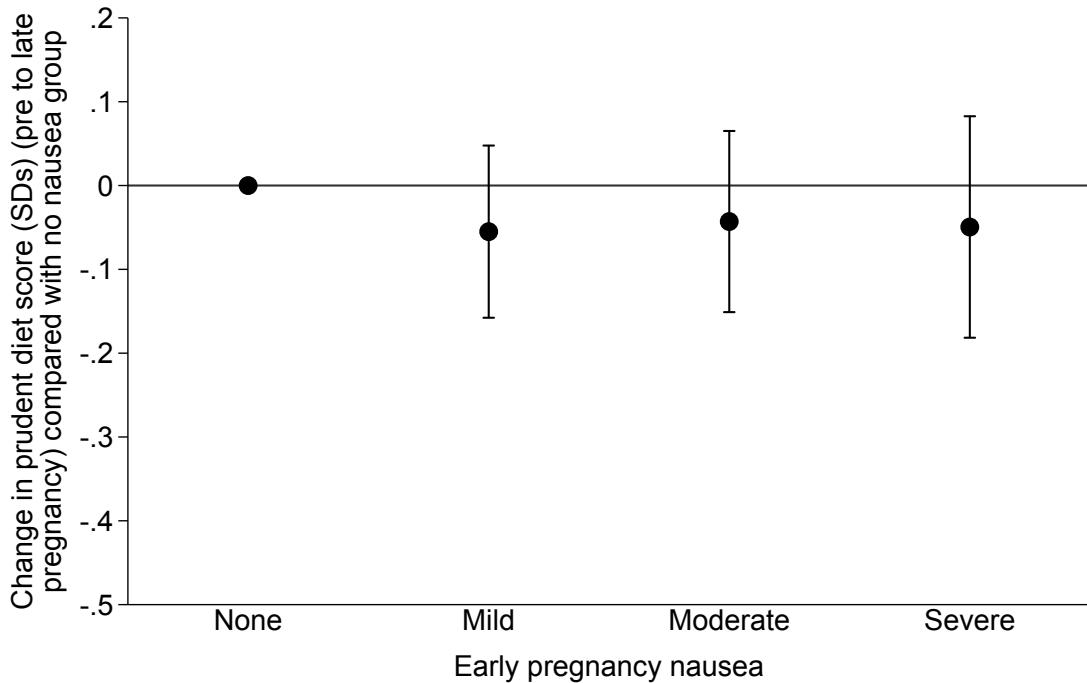

Values are adjusted difference (95% CI)

Supplement: Supplementary file 2 — Supplementary Figure 2 Change in prudent diet in late pregnancy according to experience of NVP, adjusted for confounders (n = 2026) [file MCN-13-e12389-s002.pdf]
